# Chronic non-bacterial osteomyelitis associated with psoriasis

**DOI:** 10.1093/rap/rkae052

**Published:** 2024-04-10

**Authors:** Takashi Nawata, Mako Yonezawa, Masafumi Fujinaka, Masaki Shibuya, Motoaki Sano

**Affiliations:** Department of Medicine and Clinical Science, Yamaguchi University Graduate School of Medicine, Ube, Japan; Department of Medicine and Clinical Science, Yamaguchi University Graduate School of Medicine, Ube, Japan; Department of Medicine and Clinical Science, Yamaguchi University Graduate School of Medicine, Ube, Japan; Department of Medicine and Clinical Science, Yamaguchi University Graduate School of Medicine, Ube, Japan; Department of Medicine and Clinical Science, Yamaguchi University Graduate School of Medicine, Ube, Japan

A 34-year-old Japanese man presented to our hospital with right jaw pain and trismus for 2 years (Fig. 1A). Bone scintigraphy findings revealed increased uptake in the right mandible (Fig. 1B). No other increased uptakes in the bones and joints were noted. CT revealed osteolytic lesions of the right mandible (Fig. 1C). The right mandible biopsy findings were compatible with chronic non-bacterial osteomyelitis (CNO). Psoriatic plaques in the bilateral knees and lower legs, which first appeared in adolescence, were evident (Fig. 1D). The joints were non-tender and had no signs of inflammation. Laboratory examination showed elevated serum CRP levels (6.34 mg/dl; normal range: <0.14 mg/dl). The skin biopsy findings confirmed the diagnosis of psoriasis.

**Figure 1.**
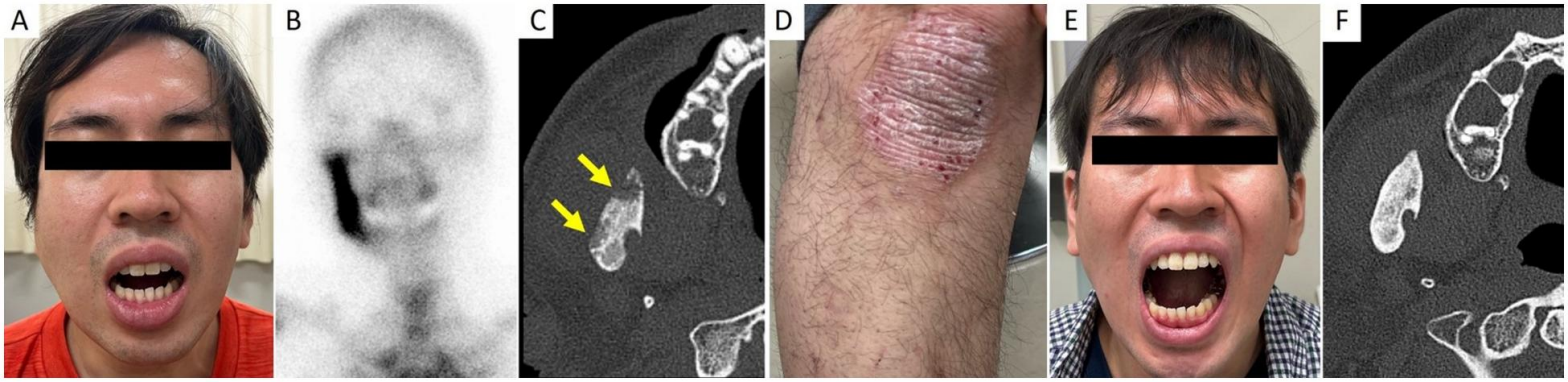
Imaging findings of the patient. **(A)** Swelling of the right jaw and trismus. **(B)** Bone scintigraphy findings show increased uptake in the right mandible. **(C)** CT reveals osteolytic lesions of right mandible (arrow). **(D)** Psoriatic plaque observed in the right knee. **(E)** Swelling of the right jaw and trismus improved after administration of certolizumab pegol (4 weeks after initiation of treatment). **(F)** CT reveals the improvement of osteolytic lesions of right mandible (5 months after initiation of treatment)

Based on the diagnosis of CNO associated with psoriasis, subcutaneous administration of certolizumab pegol (initial dose 400 mg biweekly) was initiated. After 4 weeks, the right jaw symptoms and psoriatic plaques improved (Fig. 1E). Further, the osteolytic lesions also improved after 5 months (Fig. 1F).

Few cases of CNO accompanied by psoriasis have been reported previously [1–2]. TNF-α inhibitors and bisphosphonates are used to treat CNO [1–2]. Our case suggests the usefulness of TNF-α inhibitors for both CNO and psoriasis.

## Data Availability

All the relevant data supporting the findings of this case report are available within the article.
